# Sexual dimorphism in NLR transcripts and its downstream signaling protein IL-1ꞵ in teleost *Channa punctata* (Bloch, 1793)

**DOI:** 10.1038/s41598-024-51702-7

**Published:** 2024-01-22

**Authors:** Bhawna Chuphal, Priyanka Sathoria, Umesh Rai, Brototi Roy

**Affiliations:** 1 Department of Zoology, Miranda House, University of Delhi, Delhi, 110007 India; 2 Department of Zoology, Maitreyi College, University of Delhi, Chanakyapuri, Delhi, 110021 India; 3 University of Jammu, Jammu and Kashmir, Jammu, 180006 India

**Keywords:** Immunology, Molecular biology, Zoology

## Abstract

Nucleotide-binding oligomerization domain-like receptors (NOD-like receptors or NLRs) are a family of intracellular pattern recognition receptors (PRRs) that initiates as well as regulate inflammatory responses. NLRs are characterized by a centrally located nucleotide binding domain and a leucine rich repeat domain at the C-terminal responsible for the recognition of intracellular microbe-associated molecular patterns (MAMPs) and danger-associated molecular patterns (DAMPs). In the present study in adult spotted snakehead we have investigated the sex-dependent tissue distribution of NLRs known to be associated with inflammation in teleost namely NOD1, NOD2, NLRC3, NLRC5, and NLRX1. Further, the sexual dimorphism in the expression of NLR transcript as well as the pro-inflammatory protein IL-1β was explored in fish under normal conditions, and in fish exposed to bacterial lipopolysaccharide (LPS). The NLRs show ubiquitous and constitutive expression in all the tissues. Moreover, a prominent disparity between males and females was observed in the basal expression of these genes in various tissues. The sexual dimorphism in NLR expression was also prominent when fish were exposed to LPS. Similarly, IL-1β exhibited sexual dimorphism in both normal as well as LPS-exposed fish.

## Introduction

The germ-line-encoded pattern recognition receptors (PRRs) are key molecules of the immune system that initiates as well as regulates inflammation in response to pathogens or altered internal milieu. The membrane bound PRRs include toll-like receptors (TLRs) and C-type lectin receptors (CLRs) whereas the cytosolic PRRs include retinoic acid inducible gene I (RIG-I)-like receptors (RLRs), cytosolic DNA sensors (CDSs), NOD-like receptors (NLRs) and the newly identified cytosolic absent in melanoma (AIM)-like receptors^1–4^. NLRs are characterized by centrally located nucleotide binding domain and C-terminal leucine rich repeat domain and are broadly divided into NLRA, NLRB, NLRC and NLRX1 subfamilies based on N-terminal effector binding domain. In teleosts, extensive research on NOD1 and NOD2 highlights their widespread expression patterns. Furthermore, investigations involving bacterial ligands and viruses underscore the pivotal roles played by NOD1 and NOD2 in the host's defense mechanisms against both bacterial and viral infections^5^. NLRC3 and NLRC5 act as positive regulators of immune responses in teleosts^6^. Teleost NLRX1 has been reported critical in controlling innate immune responses and oxidative stress at mitochondria^7^. NLRs act in tandem with other cytosolic and transmembrane PRRs to sense intracellular pathogens/pathogenic ligands, thereby activating the downstream inflammatory pathways ultimately leading to release of pro-inflammatory cytokines involved in inflammatory responses^8^.

The inflammatory responses in mammals are reported to have dramatic gender-based differences, thereby leading to differential sex-based susceptibility to infection, sepsis, and autoimmune diseases^9^. Similar to mammals, a few studies in teleost also report sex-dependent differential immune responses. For example, sex-related differences in catalase (CAT) and superoxide dismutase (SOD) activity is observed in zebrafish wherein the females reported higher CAT activity, and males showed higher SOD activity^10^. On the contrary, in Chapultepec splitfin, male showed higher lipid peroxidation (LPOX), SOD, and CAT activity as compared to female counterparts^11^. In addition, the immune response in males and females have also been observed to respond differentially to bacterial challenges/ligands or physiological hormonal milieu. For instance, in response to lipopolysaccharide (LPS), the expression of suppressor of cytokine signaling (SOCS) was enhanced in male head kidney as compared to female yellow perch^12^. Similarly, in goldfish, hCG exposure increased the expression of cytokines such as TNF-α in female spleen, liver and head kidney as compared to male counterparts^13^. In rainbow trout, after surgical tag implantation, male counterparts showed higher cytokine levels than mature females^14^.

Interestingly, despite their important role in inflammation, till date no studies have addressed the differential sex-dependent expression of NLRs in any vertebrate group. Given this, the present study was undertaken to investigate the sexual dimorphism in NLR expression in various tissues of teleost *Channa punctata*. Further, we also explored how the sexually dimorphic expression of NLRs and the proinflammatory effector molecule, IL-1β was altered in response to challenge by bacterial ligand, lipopolysaccharide (LPS). The spotted snakehead, *Channa punctata* (Bloch, 1793), breeds in flooded rivers and ponds during south-west and north-east monsoons^15^ and is distributed throughout the South-East Asian countries. The IUCN has listed it as low-risk near threatened fish species in India^16^. This economically important fish is well known for its high nutritive value, taste, and medicinal qualities and is recommended as a diet during convalescence^17^. The reproductive cycle of spotted snakehead is divided into 4 phases viz: regressed (December-March), preparatory (April-June), spawning (July–August) and post spawning (September–November)^18^. Being a seasonal breeder, the immune system of the fish is exposed to seasonally changing reproductive hormonal milieu wherein the preparatory phase exhibits high levels of sex steroids^18–21^ Hence to study the sex-dependent NLR and IL-1β expression, the experiments were performed during the preparatory phase when the immune system of the fish is exposed to high levels of sex steroids.

## Results

### Tissue- and sex-dependent expression of NLRs

**Figure 1:**
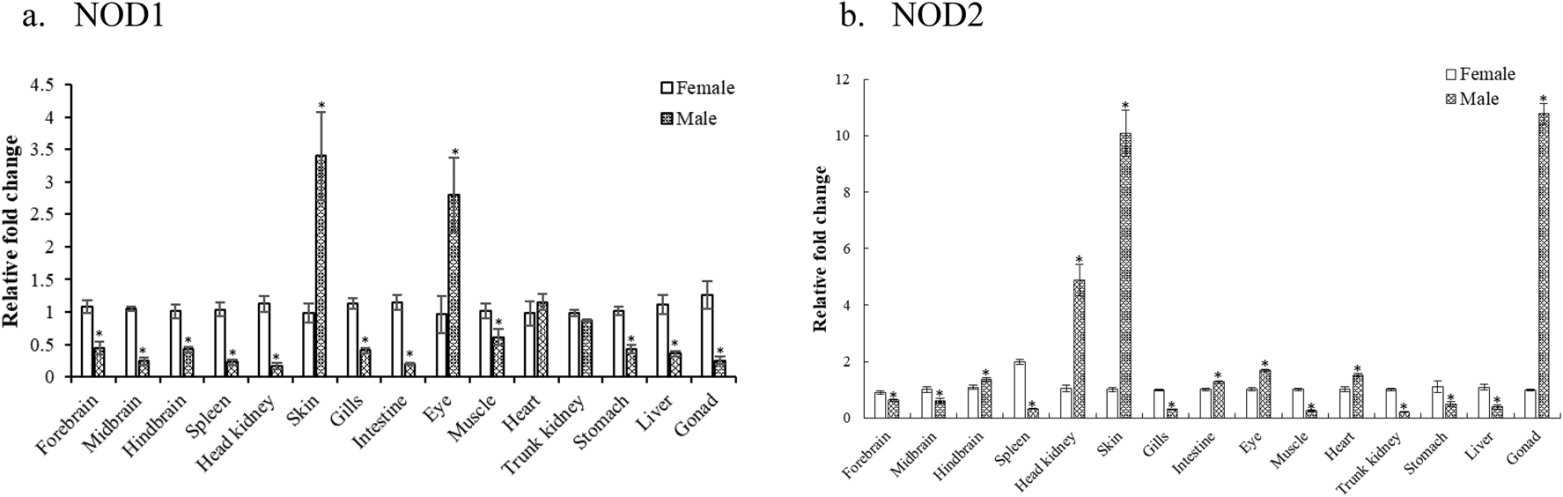
Variation in expression of (**a**) ssNOD1, (**b**) ssNOD2 in various tissues of both male and female *C*. *punctata* during preparatory phase. Tissues namely, anterior, mid and posterior brain, head kidney, spleen, skin, gills, eye, muscle, heart, trunk kidney, stomach, liver, intestine and gonad from both male and female (N = 8) were excised out. qPCR was carried out and the data was normalized using *18S rRNA* and β*-actin* genes as reference. The relative fold change was calculated following the 2^−∆∆Ct^ method with female as reference. Student’s unpaired t-test was employed to calculate significant difference (*p* < 0.05, female vs. male) for each tissue. Data is shown as a fold change in gene expression (Mean ± SEM). Asterisks indicate significant difference (*p* < 0.05, female vs male).

**Figure 2:**
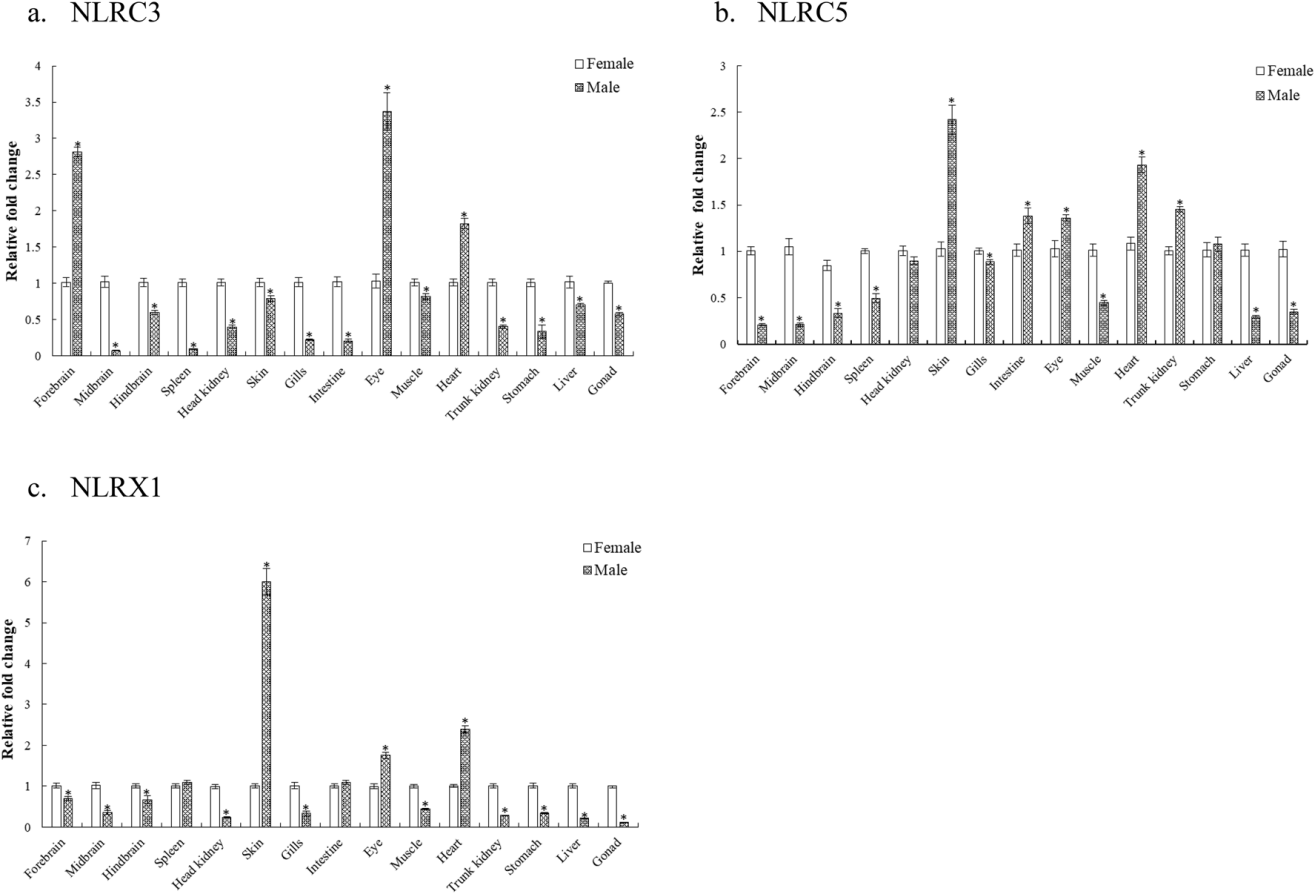
Tissue-dependent variation in expression of (**a**) ssNLRC3, (**b**) ssNLRC5 and (**c**) ssNLRX1 in both male and female *C*. *punctata*. Tissues of interest, namely, brain (anterior, mid and posterior), immune organs (head kidney, spleen, skin, gills and intestine), eye, muscle, heart, trunk kidney, stomach, liver, and gonad from male and female were excised out during preparatory phase. For gene expression analysis, qPCR was performed with two technical replicates. After normalizing the data with *18S rRNA* and β*-actin* reference genes, female was set as a control for calculating fold change data. Student’s unpaired t-test was employed to calculate significant difference for each tissue. Data is shown as fold change in gene expression (Mean ± SEM, N = 8 for each sex). Asterisks indicate significant difference (*p* < 0.05, female vs. male).

Quantitative expression analysis of spotted snakehead (ss) NOD1, NOD2, NLRC3, NLRC5 and NLRX1 genes revealed ubiquitous and constitutive expression in all the tissues examined thereby validating their role in innate immunity. In general, most of the tissues examined in the current study showed sexually dimorphic expression of NLRs during the preparatory phase (Student’s unpaired *t*-test, *p* < 0.05, Fig. 1, 2).

#### ssNOD1 and ssNOD2

The expression levels of ssNOD1 was relatively lower (*p* < 0.0001) in male as compared to female in case of brain (forebrain, midbrain and hindbrain), immune organs such as head kidney, spleen, gills, intestine, gonads, muscle, stomach and liver. Skin and eye on the contrary exhibited higher expression in males than in females (Fig. 1a). Tissues such as heart and trunk kidney, however, did not show any sex-related difference in the expression of NOD1. Regarding ssNOD2 expression, sexual dimorphism was observed with significantly higher expression (*p* < 0.05) in forebrain, midbrain, muscle, trunk kidney, stomach, liver and immune organs namely spleen and gills in case of female while in case of male, higher expression (*p* < 0.05) was noted in hindbrain, head kidney, skin, intestine, eye, heart as well as gonads (Fig. 1b).

#### ssNLRC3, ssNLRC5 and ssNLRX1

Sexually dimorphic expression of ssNLRC3 transcript level was observed in all the tissues studied wherein forebrain, eye and heart had comparatively higher expression level (*p* < 0.0001) in male than female whereas expression was relatively lesser (*p* < 0.05) as compared to females in all the other tissues (Fig. 2a). Expression analysis of ssNLRC5 revealed sex-related differential expression in which brain, spleen, gills, muscle, liver and gonads showed more expression (*p* < 0.05) in case of females as compared to males whereas relatively higher expression (*p* < 0.05) in male was seen in skin, intestine, eye, heart, trunk kidney. Interestingly, head kidney and stomach did not show any significant difference in NLRC5 transcript level (Fig. 2b). The expression of mitochondrially located receptor, ssNLRX1 was found to be relatively lesser in skin, eye and heart in females as compared to males while the expression was higher (*p* < 0.05) in rest of the tissues namely brain, head kidney, gills, muscle, trunk kidney, stomach, liver and gonads. The expression was not significant in case of spleen and intestine (Fig. 2c).

### Effect of LPS on NLR expression

**Figure 3:**
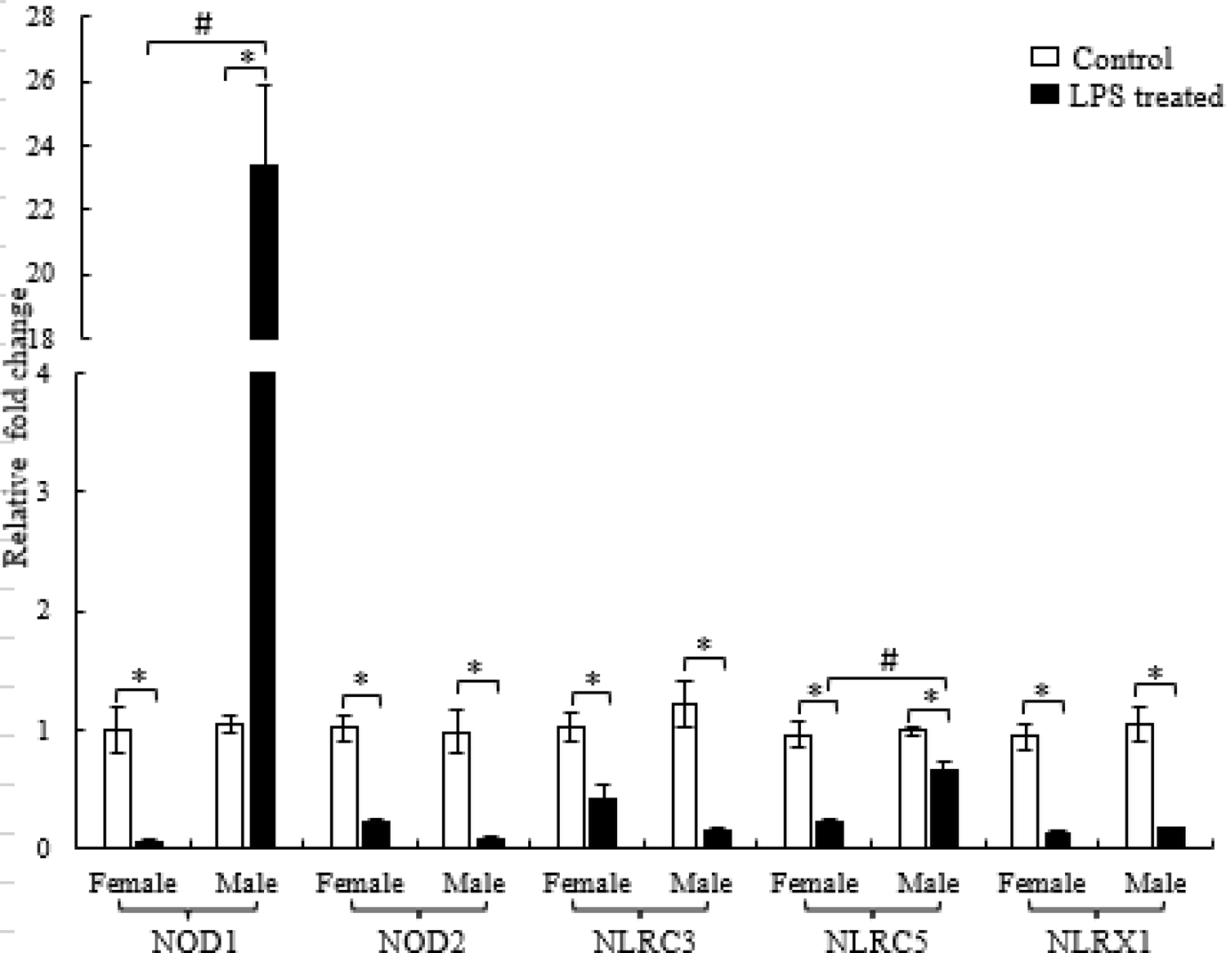
Differential expression of splenic NLRs in male and female *C*. *punctata* in response to LPS. Differential expression of NLRs in lymphoid organ, spleen of male and female *C*. *punctata* was studied in response to LPS injection or 1X PBS (control) for 6 h. The data was normalized with *18S rRNA* and β*-actin* genes and control was set as a reference for calculating fold change data. Significant difference between the groups was calculated employing two-way analysis of variance followed by Tukey’s range test. White bars represent male whereas black bars represent female. Data is represented as fold change in gene expression (Mean ± SEM, N = 8 for each group). ‘*’ indicate significant difference (*p* < 0.05, control vs LPS treated) and ‘#’ indicates a significant difference between male and female.

The NLR expression was significantly altered following a challenge with bacterial lipopolysaccharide. Moreover, the effect of LPS was sex-dependent. In spleen, the expression of ssNOD1 in male was significantly elevated in the LPS-treated group (*p* < 0.0001) as compared to the control group. On the contrary, in female, significant downregulation was observed in NOD1 transcript level in the LPS-treated group (*p* < 0.05). In case of ssNOD2, female as well as male showed significant decline in its expression in the LPS-treated group as compared to control group (*p* < 0.01) (Fig. 3).

The differential expression of ssNLRC3, ssNLRC5 and ssNLRX1 followed a similar pattern wherein a significant decrease in the gene expression was observed in the LPS-treated group as compared to control group in case of both male and female. However, in case of NLRC5, the downregulation is more pronounced in female as compared to male (*p* < 0.01) (Fig. 3).

#### Effect of LPS on IL-1β expression

**Figure 4:**
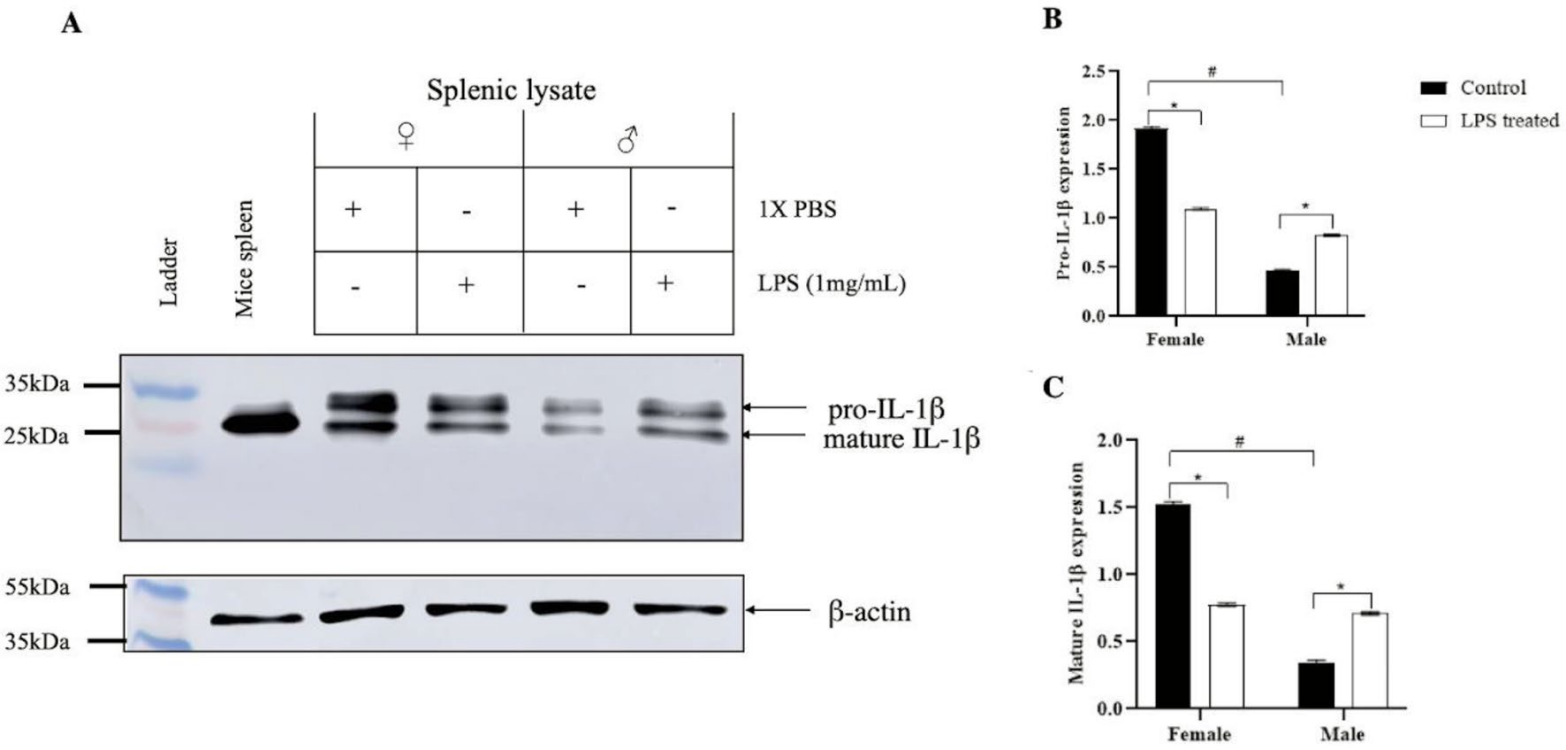
Differential expression of IL-1β in splenic lysate of male and female *C*. *punctata* in response to lipopolysaccharide (LPS). Male and female spotted snakeheads received injections of LPS (1 mg/mL) or 1 × PBS (control) for 6 h. (**A**) Total splenic protein was isolated and analyzed using SDS-PAGE and Western blotting using anti-IL-1β antibody. β-actin-antibody (approximately 42 kDa) was used as a loading control. Quantitative estimation of image using Image J software was done for (**B**) precursor pro-IL-1β protein expression and (**C**) mature IL-1β protein expression using Microsoft Excel, where β-actin was used for normalization and statistical significance was analyzed using GraphPad Prism8 software, where two-way ANOVA was applied followed by Tukey’s range test (*p* < 0.05) for any significant change. These results are representative of three independent experiments. ‘*’ indicate significant difference (*p* < 0.05, control vs LPS treated) and ‘#’ indicates a significant difference between male and female.

The precursor and mature form of IL-1β in spotted snakehead is being identified for the first time wherein the splenic cell lysate showed the precursor sspro-IL-1β of 30 kDa, along with a processed form of approximately 24 kDa. The expression was significantly altered following a challenge with bacterial lipopolysaccharide and effect was sex-dependent. In case of the control group, male showed weaker production of precursor as well as processed IL-1β as compared to female. However, when injected with LPS, the expression of both pro-IL-1β as well as processed IL-1β enhanced significantly in males. On the contrary, in case of females, LPS downregulated the production of pro-IL 1β (Fig. 4).

## Discussion

NOD-like receptors play a critical role in inflammatory responses. The expression of NLR genes have been previously studied in teleosts wherein the expression of these receptors, although ubiquitous, showed tissue-dependent variation depending upon fish species^5,6^. Similarly, in the present study, ssNOD1, ssNOD2, ssNLRC3, ssNLRC5 and ssNLRX1 transcripts were detected in all the tissues studied, reflecting the ubiquitous distribution as well as constitutive expression. The ubiquitous and constitutive expression of NLRs reflects their role as an intracellular sentinel that aids in combating invading pathogens or destructive self-proteins. It was interesting to note that in addition to the immune tissues of *Channa punctata*, the expression level of most of these receptors was high in gonads and the different regions of the brain implicating their role in reproduction and nervous functions. In fact, in the case of mammals, the importance of transmembrane PRRs in reproduction^22^ and nervous functions such as appetite and control of body temperature^23^ are well-defined, though no such reports are available in teleosts.

In mammals, sexual dimorphism has been described in both innate^9^ and adaptive immune responses^9^. It has been reported that the TLR expression differs between sexes thus influencing the strength of TLR-dependent responses^24^. Differences in immune responses between males and females have been also documented in other species such as arthropods, reptiles, and birds wherein innate as well as adaptive immune responses were reported to be generally lower in male counterparts^25,26^. However, no study is available on sex-related differential expression of NOD-like receptors and their downstream effector molecules across vertebrates, despite the important role played by them in inducing and regulating inflammation. The present study in spotted snakehead reports the sexually dimorphic expression of NLRs under normal conditions and in response to lipopolysaccharide (LPS) injection. The NLR expression in the eye and skin of male fish was seen higher as compared to the female counterparts. The higher expression of NLR in the eye and skin in male *Channa punctata* might contribute to a more robust ocular and mucosal immunity as has been described earlier^27–30^. However, the NLR expression in most other tissues in the case of males was lesser as compared to females indicating a better systemic immunity under normal conditions in female *Channa punctata*. The sexually dimorphic expression may be attributed to Bateman’s principle which suggests that males and females invest differentially in immune system parameters with females maximizing fitness by lengthening their lifespan through greater investment in immune defenses^31^. A few authors have supported this principle stating that as compared to females, males might invest fewer resources towards immunity and more towards mating success by allocating it to develop sexually selected traits^32–34^.

LPS treatment led to decreased transcription of all the NLRs except ssNOD1 in both the sexes. This phenomenon aligns with previous findings in murine liver cells where LPS substantially inhibited many immune mediators such as IFN-γ from activated T cells and diminished surface expression of MHC class II, CD80, and CD86^35^. In addition, a study in periparturient dairy cows reported downregulation of NLRs in neutrophils^36^. Since inflammation is an energetically costly physiological process, it has been suggested that the downregulation of some immune responses against pathogens might have evolved to temper the detrimental effects of inflammation on hosts^35^. Similarly the suppression of NOD2, NLRC3, NLRC5 and NLRX1 transcripts upon LPS induction in *Channa punctata* might serve to attenuate excessive inflammatory responses, thereby contributing to the maintenance of tissue homeostasis crucial for cell survival.

In case of males, the expression of NOD1 was dramatically enhanced in response to LPS, whereas, in females, ssNOD1 expression was suppressed. NOD1 is implicated in the recognition of the bacterial component, LPS, and activation of the NF-κB signal pathway resulting in the expression of inflammatory cytokines^37,38^ along with the processing of pro-IL-1β^38^. Hence, based on the overexpression of ssNOD1 in response to LPS in male *Channa punctata*, it may be possible that males exhibit increased expression as well as the processing of IL-1β in contrast to females where significant downregulation of NOD1 expression was seen. Among the several proinflammatory cytokines implicated during infection and immune challenges, IL-1β is quintessential as it is involved in acute and chronic inflammation^39^. In addition, the coordinated regulation of circulating mature IL-1β is required for maintaining homeostasis^40^. In teleosts, IL-1β has been identified in several species^41–51^ and the processing of pro-IL-1β has been demonstrated^40,52,53^. However, the size of the precursor pro-IL-1β as well as the mature form differs considerably in different teleostean species^53,54^. The current study for the first time reports IL-1β precursor and mature form in *Channa punctata*. pro-IL-1β identified by Western blotting with anti-IL-1β antibody was approximately 30 kDa and the mature form of IL-1β was approximately 24 kDa. A similar size of mature IL-1β was also observed in rainbow trout^54^. Interestingly, in spotted snakehead, sexual dimorphism was also observed in the expression level of both precursor and mature IL-1β being higher in females under normal conditions. LPS injection also showed diverse effect on IL-1β expression in males and the effect could be correlated with the NOD1 expression. Similar results have been previously reported wherein LPS-challenged human serum and murine-derived macrophages had higher levels of cytokines including IL-1β in males as compared to females^55,56^.

Thus, our results raise a possibility that although females have a more robust surveillance system under normal conditions, males might be mounting a more aggressive inflammatory response during bacterial infection in teleost. However, the implication of the sexually dimorphic cytokine production on the survival of fish during infection is yet to be deciphered. Considering the threat faced by intensive aquaculture practices due to frequent disease outbreaks^57–60^, it would be of interest to explore the significance and implication of the sexually dimorphic inflammatory responses in teleost. The present study might provide a prospect for sex-dependent differential development of fish vaccines and the judicious use of immunostimulants against bacterial and viral diseases.

## Conclusion

This study for the first time reported the sex-dependent differential expression of NOD-like receptors, an important component of both innate as well as adaptive immunity. The NLR transcript expression as well as IL-1β protein expression in female *C*. *punctata* was higher as compared to males. This might reflect their higher investment in immunity to increase their lifespan. Interestingly, when exposed to bacterial lipopolysaccharide, increased expression of NOD1, widely reported to initiate and regulate inflammation was observed in males. Similar overexpression of effector protein IL-1β was also observed in males, thus suggesting a robust immune response against pathogens in males as compared to females. Further studies to understand how these sex-dependent differential inflammatory reactions impact the response of fishes to bacterial infections as well as vaccinations would have great significance in aquaculture.

## Materials and methods

### Procurement and maintenance of fish

In the present study, adult spotted snakehead *Channa punctata* was used as an experimental model. Males are identified by the presence of a circular genital opening whereas in females the opening is elongated. Multiple tiny well-defined black dots are present randomly on the lateral side of the male fish, whereas in case of females the dots are diffused and larger in size (Supplementary file S1). Fish (80–100 g) procured from wild population in and around National Capital Region of Delhi, India were stocked in tanks [dimension: 74 cm (L) × 34 cm (B) × 32 cm (H), Sintex] containing dechlorinated fresh water (15 fish/tank containing 45 L water) which was changed on alternate days under 12 L:12 D light regimen at 25 ± 2 °C. Acclimation was carried out for 3 weeks during which water temperature varied from 24 to 26 °C. Water used in the experimental setup had a pH of 7.5, conductivity of 367µS/cm, total dissolved solids (TDS) value was 256 ppm along with 193 ppm salinity and 8.2 mg/L of dissolved oxygen. After completion of the experiment, fish were sacrificed with an overdose of 2-phenoxyethanol (5 ml L-1, Sisco Research Laboratories, Mumbai, India). The protocol has been approved by the Institutional Animal Ethics Committee (DU/ZOOL/IAEC-R/2019/10), Department of Zoology, University of Delhi and all the methods were performed in accordance with the relevant guidelines and regulations of the IAEC. The studies involving the live animals follow the recommendations in the ARRIVE guidelines.

### Tissue- and sex-dependent expression of NLRs

Female and male *C. punctata* during the preparatory phase (April-June) were sacrificed (N = 8 per sex). Different tissues including brain (anterior, middle and posterior regions), eye, heart, liver, stomach, muscle, gonads, and lymphoid organs namely head kidney, spleen, skin, gills, intestine and trunk kidney were dissected out. For the expression analysis at gene level, tissues were washed in 1X PBS, zap frozen in liquid nitrogen and stored at − 80 °C until processed further. The expression of NLRs in each tissue of female was compared with respective gene expression in the same tissue of male.

### Effect of LPS on the expression of NLRs

Adult male spotted snakeheads were separated into two groups, namely experimental and control group (N = 8 for each group). The experimental group was injected intraperitoneally with 200µL of LPS (1 mg/mL; derived from *Escherichia coli* O111:B4, Sigma) and the control group received equal volume of 1X PBS. Similar experimental and control groups were made for females (N = 8 for each group). Six hours after injection, the spleen was collected, zap frozen in liquid nitrogen and stored at − 80 °C until use. The time was decided based on a pilot experiment performed with LPS (unpublished).

### Total RNA extraction and cDNA synthesis

Total RNA was extracted in TRIzol reagent following the manufacturer's protocol (Invitrogen). RNA levels were quantified using a Nanodrop (ND-1000, NanoDropTechnologies, USA) and band intensities of *28S* and *18S rRNA* were observed on 1% agarose gel for assessment of integrity. For cDNA preparation, 1 μg total RNA was treated with enzyme DNase I to remove genomic contamination. The treated samples were then processed using avian myeloblastosis virus (AMV) reverse transcriptase for reverse transcriptional synthesis of single-strand cDNA following manufacturer’s specifications (Cat# K1622, Thermo Scientific, USA) in a thermocycler. The conditions for RT-PCR were as follows: initial denaturation (95 °C for 3 min), 30 cycles each for denaturation at 95 °C for 30 s, annealing for 30 s (at gene specific primer temperature; Table 1) and extension at 72 °C for 45 s, followed by final extension of 10 min at 72 °C. The synthesized cDNA was checked using the primers of the *18S rRNA* gene^61^.

**Table 1:** List of primers used for semi-quantitative polymerase chain reaction (RT-PCR) and qPCR.

S/No	Gene	Type of primer		Primer sequence	Annealing temperature (°C)	Reference
1	NOD1	Semi-quantitative	Forward Reverse	5’ TTCCTCCACATCACACTCCA 3’ 5’ CCCACATTCCCACATCAAA 3’	58	^62^
		qPCR	Forward Reverse	5’ GACAACAACAACATCAGCGACT 3’ 5’ TAAAGCCCCAAAACCTCCA 3’	60	
2	NOD2	Semi-quantitative	Forward Reverse	5’ CAGTGTTTCTTTGCTGCTCTG 3’ 5’ GCTTTGCTCCTTCTGATGTTATG 3’	57	^63^
		qPCR	Forward Reverse	5’ GCTGCTCTCCTGCTATGATT 3’ 5’ TCCACAGGTTGAGGGATAGA 3’	60	
3	NLRC3	Semi-quantitative	Forward Reverse	5’ ACAATTGGTTCTAAAGGTGC 3’ 5’ ATCCATTCCCAGAGAGTTC 3’	56.7	^63^
		qPCR	Forward Reverse	5’ ACACTCTGCTTTCTCTCCA 3’ 5’ GTTCTTTGCTCCCTCCAC 3’	56.7	
4	NLRC5	Semi-quantitative	Forward Reverse	5’ GCCAAATCTCACTTCTCTCAG 3’ 5’ CATCCCATACCCACATCCTG 3’	60.5	^63^
		qPCR	Forward Reverse	5’ TTAGCCTGGAGAGCCTATGT 3’ 5’ CTTGAGGCTGCGTAGAGATTT 3’	58.2	
5	NLRX1	Semi-quantitative	Forward Reverse	5’ GGTGAACCTGCTGAGGAAATA 3’ 5’ CTCTTCGTCCGTCTTGGTTT 3’	56.5	^63^
		qPCR	Forward Reverse	5’CTGCTTCCTCCCGTCTTATTG 3’ 5’ GCCTGAGGAAACTGGTGTAAA 3’	60.1	
6	β-actin	qPCR	Forward Reverse	5’ GGGAGTGATGGTTGGTATGG 3’ 5’ TGGGTATTTCAGGGTCAGGA 3’	58	Current study
7	18S rRNA	qPCR	Forward Reverse	5’ CTGAACTGGGGCCATGATT 3’ 5’ CTTTCGCTTTCGTCCGTCT 3’	57.4	^61^

### Gene quantification by quantitative polymerase chain reaction (qPCR)

In order to quantitate target mRNA transcripts of NLRs, partially validated sequences of NOD1 , NOD2, NLRC3, NLRC5 and NLRX1 were used for designing qPCR primers (Table 1) . Further, qPCR was carried out in Applied Biosystems ViiA 7 Real-time PCR system (Thermo Fisher Scientific, Massachusetts, USA). The reaction mixture contained 5 μl SYBR Green master mix (Applied Biosystems, Massachusetts, USA), 0.5 μl forward and reverse gene-specific primers each, 1 μl cDNA and 3 μl nuclease-free water. Thermocycling conditions were as follows: initial denaturation at 95 °C for 10 min, 40 cycles of denaturation at 95 °C for 10 s and annealing at specific temperature for 1 min. The specificity of a single product and absence of primer dimer were confirmed at the end of each cycle by melting curve analysis. Samples were run in duplicate and no template control (NTC) was run with each reaction.

### Effect of LPS on the expression of interleukin-1β

#### Preparation of splenic extract

A part of the splenic tissue (50 mg per group per sex) collected from the above experiment was used for preparing the extract (N = 3). Tissues were homogenized in chilled RIPA (radioimmunoprecipitation assay) lysis buffer along with 100 mM PMSF (phenylmethylsulfonyl fluoride) and centrifuged at 10,000 g for 25 min. The supernatant was collected and stored at − 80 °C for further use.

#### Western blot

Protein concentration was estimated by Lowry et al.^64^ method, using bovine serum albumin (BSA) as standard. Splenic lysate (60 µg/well) was resolved in 15% SDS-PAGE and transferred onto the nitrocellulose membrane in the transfer buffer (25 mM Tris, 193 mM glycine, 20% methanol, pH 8.5). After transfer, nitrocellulose membrane was incubated with the 5% BSA blocking solution for 1 h and subsequently incubated with the primary antibody dilution 1:1000, anti-mouse IL-1β (BB-AB0165) and anti-mouse β-actin (sc-47778, Santa-cruz) overnight at 4 °C. The membrane was washed 3 times with TBST (50 mM Tris, 150 mM NaCl, 0.1% Tween-20, pH 7.6) and further incubated with goat anti-mouse IgG-HRP (Horseradish peroxidase) conjugated antibody (dilution 1:1000) for 1.5 h and washed 3 × with TBST. The bands were developed using Luminata™ Crescendo Western HRP substrate (Millipore Corporation) and observed under the luminescent image analyzer amersham imager-600 (GE/Biosciences AB). The quantitative densitometric analysis of the bands was performed using ImageJ software.

### Statistical analyses

The relative fold change was calculated following the 2^−∆∆Ct^ method^65^. To analyze expression values in each sample of target tissues, ribosomal 18S RNA *(18S rRNA*) and *β-actin* were used as reference genes. The mean Ct value of the tissue showing minimum expression for respective genes and control was set as reference for tissue- dependent and in vivo LPS-induced ΔΔCt gene expression analysis, respectively. For tissue- and sex-dependent expression of NLRs, Student’s unpaired *t*-test (*p* < 0.05) was employed. For differential expression dependent on sex (female vs. male) as well as in vivo LPS treatment, two-way analysis of variance (ANOVA) was applied followed by Tukey’s range test (*p* < 0.05) for any significant change in gene expression. Data shown in the result section is represented as mean ± standard error of mean (SEM). The software used for statistical analyses was GraphPad Prism8 software (GraphPad Software, La Jolla, CA). For quantitative estimation of protein, the data analysis was performed using Image J software and Microsoft Excel, β-actin was used for normalization and statistical significance was analyzed using GraphPad Prism8 software (GraphPad Software, La Jolla, CA), where two-way ANOVA was applied followed by Tukey’s range test (*p* < 0.05) for any significant change in protein expression.

### Supplementary Information

Supplementary Information 1.

Supplementary Information 2.

Supplementary Information 3.

## Data Availability

The datasets presented in this study can be found in online repositories. The name of the repository/repositories and accession number(s) can be found below: https://www.ncbi.nlm.nih.gov/, MK328029; https://www.ncbi.nlm.nih.gov/, MK395360; https://www.ncbi.nlm.nih.gov/, MK395361; https://www.ncbi.nlm.nih.gov/, MK328031; https://www.ncbi.nlm.nih.gov/, MK328030.
