# Supplementary material for: Sexual dimorphism in NLR transcripts and its downstream signaling protein IL-1ꞵ in teleost Channa punctata (Bloch, 1793)
Source: Sci Rep. 2024 Jan 22;14:1923. doi: 10.1038/s41598-024-51702-7 (PMC10803744; doi:10.1038/s41598-024-51702-7)
Supplement: Supplementary file 2 — Supplementary Information 2. [file 41598_2024_51702_MOESM2_ESM.docx]

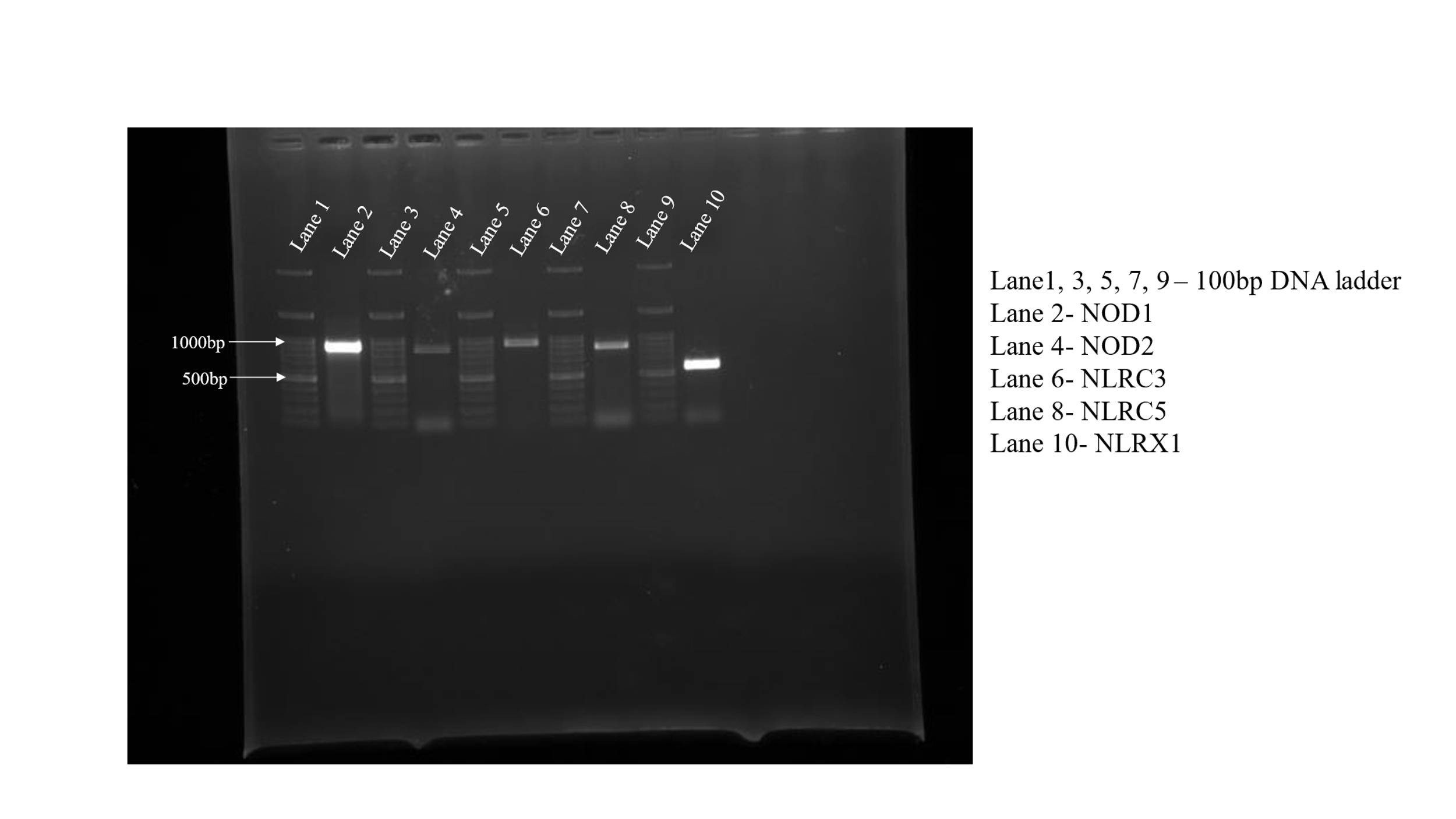


**Supplementary Figure**: Gel image of semi-quantitative primers (NOD1, NOD2, NLRC3, NLRC5 and NLRX1)


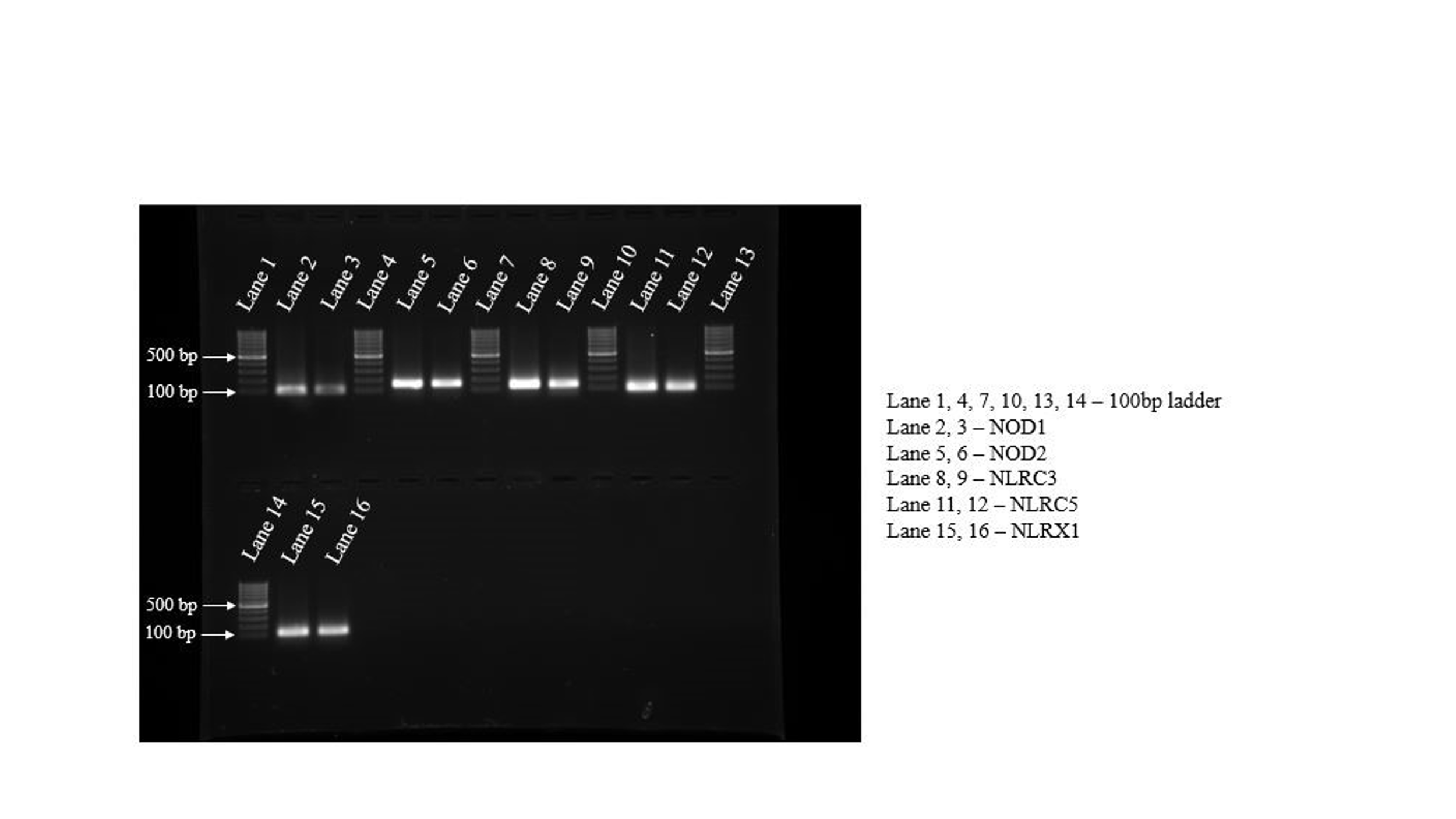


**Supplementary Figure**: Gel image of qPCR primers (NOD1, NOD2, NLRC3, NLRC5 and NLRX1)
