# Supplementary material for: Sexual dimorphism in NLR transcripts and its downstream signaling protein IL-1ꞵ in teleost Channa punctata (Bloch, 1793)
Source: Sci Rep. 2024 Jan 22;14:1923. doi: 10.1038/s41598-024-51702-7 (PMC10803744; doi:10.1038/s41598-024-51702-7)
Supplement: Supplementary file 3 — Supplementary Information 3. [file 41598_2024_51702_MOESM3_ESM.docx]

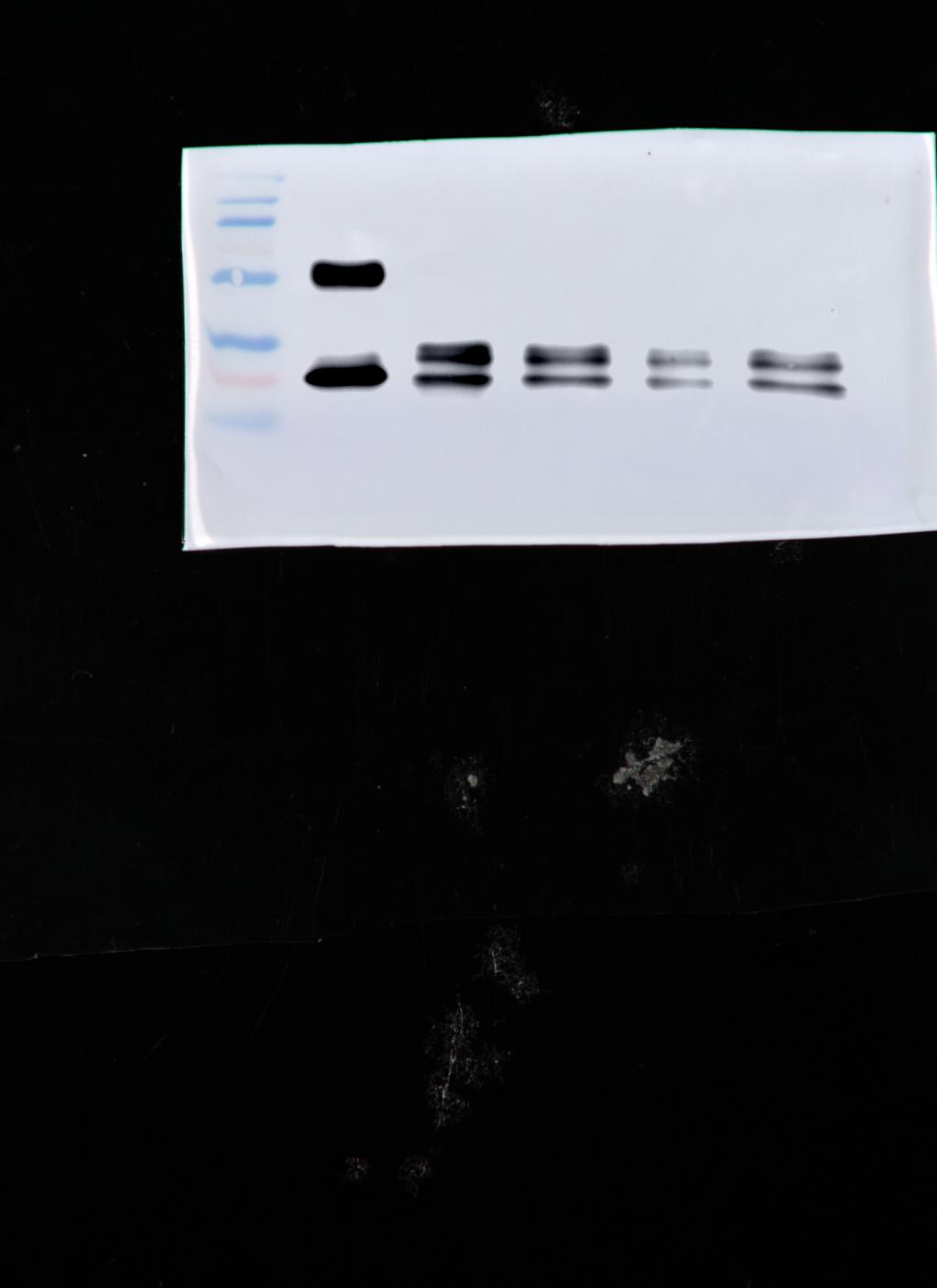

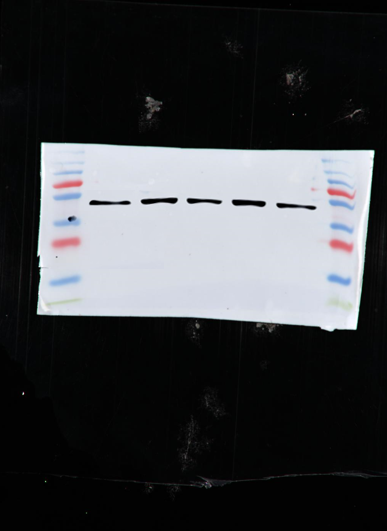


**Supplementary Figure S3:** Differential expression of IL-1β in splenic lysate of male and female *C*. *punctata* in response to lipopolysaccharide (LPS)
